# Antidiabetic Potential of Plants from the Caribbean Basin

**DOI:** 10.3390/plants11101360

**Published:** 2022-05-20

**Authors:** Vanessa Méril-Mamert, Alejandro Ponce-Mora, Muriel Sylvestre, Genica Lawrence, Eloy Bejarano, Gerardo Cebrián-Torrejón

**Affiliations:** 1 Laboratoire COVACHIM-M2E EA 3592, Université des Antilles, CEDEX, 97157 Pointe-à-Pitre, France; vanessa.mamert@etu.univ-antilles.fr (V.M.-M.); muriel.sylvestre@univ-antilles.fr (M.S.); genica.lawrence@univ-antilles.fr (G.L.); 2 Department of Biomedical Sciences, School of Health Sciences and Veterinary, Universidad Cardenal Herrera-CEU, CEU Universities, 46113 Moncada, Spain; alejandro.poncemora1@uchceu.es

**Keywords:** diabetes mellitus, phytocompounds, hyperglycemia, glycative stress

## Abstract

Diabetes mellitus (DM) is a group of metabolic disorders characterized by hyperglycemia, insulin insufficiency or insulin resistance, and many issues, including vascular complications, glycative stress and lipid metabolism dysregulation. Natural products from plants with antihyperglycemic, hypolipidemic, pancreatic protective, antioxidative, and insulin-like properties complement conventional treatments. Throughout this review, we summarize the current status of knowledge of plants from the Caribbean basin traditionally used to manage DM and treat its sequelae. Seven plants were chosen due to their use in Caribbean folk medicine. We summarize the antidiabetic properties of each species, exploring the pharmacological mechanisms related to their antidiabetic effect reported in vitro and in vivo. We propose the Caribbean flora as a source of innovative bioactive phytocompounds to treat and prevent DM and DM-associated complications.

## 1. Introduction

Diabetes mellitus (DM) is a chronic disturbance that occurs when the hypoglycemic hormone insulin is not released in adequate amounts or when its activity is ineffective due to metabolic resistance [1,2]. Type 1 diabetes (T1D) generally begins in puberty and is an autoimmune condition in which the loss of β cells from the pancreas leads to insufficient insulin production [1]. In type 2 diabetes (T2D), the pancreas can produce insulin, but cells from different organs (fat, liver and muscle) do not respond properly to this hormone [2]. T2D accounts for 90 percent of incidence and usually begins in adulthood [3].

DM affects a large number of people, especially in low-and middle-income countries. According to the World Health Organization (WHO), about 422 million people worldwide have DM, and 1.6 million deaths are directly linked to DM or DM-related complications [4]. Furthermore, its prevalence is expected to increase, reaching 578 million people by 2030 and 700 million people by 2045 [5]. In particular, the Caribbean region is one of the fast-growing regions in DM prevalence [6]. The economic cost directly and indirectly associated with DM management is expected to increase worldwide. It will represent a challenging burden to the health system financing of countries with a high prevalence of DM, such as the Caribbean and Latin American countries [7]. 

DM is a disease of metabolic dysregulation that results in the buildup of aberrant sugar levels in the bloodstream. At the molecular level, chronic hyperglycemia leads to the accumulation of toxic advanced glycation end products (AGEs). AGEs are a diverse array of compounds formed through a non-enzymatic reaction known as glycation in which sugars or sugar metabolites attach to different biomolecules, such as proteins, impairing their function. Given that glycation is a sugar concentration-dependent reaction, high levels of glycative stress are a key pathophysiological feature in DM, being the monitorization of glycated hemoglobin A1c (HbA1c) and the most common blood test to diagnose DM [8,9]. AGE accumulation toxicity is etiologically related to multiple diabetic complications, both microvascular (retinopathy, neuropathy, and nephropathy) and macrovascular (dyslipidemia, hypertension, stroke, and myocardial infarction). Thus, DM increases the risk of several health debilities, including fatigue, poor wound healing, or chronic organ damage [10]. 

Conventional anti-DM therapies include lifestyle modifications (nutrition (e.g., low-carbohydrate diets), exercise, and weight loss), oral pharmacological agents, and subcutaneous pharmaceutical insulin [11]. Oral antidiabetic agents are used as monotherapy or in combination [12] for improved blood glucose control. As a result of this polypharmacy strategy, there are multiple possible side effects described: dizziness, constipation, nausea, diarrhea, headaches, vomiting, indigestion, loss of appetite, increased sweating, weight gain, skin reactions, and hypoglycemia [13]. Many people with DM choose to combine natural products or plant extracts with their medication to mitigate these side effects. This combination might reduce their need for medication and prevent complications [14]. Thus, the usage of herbal therapies is becoming increasingly important because of their effectiveness, limiting side effects, convenient access, and fair prices. It is estimated that the prevalence of the use of plants in people with DM is increasing worldwide in ranges between 30 and 57% [15]. In this context, DM patients use 1.6 times more complementary and herbal medicine than non-diabetics [16]. In addition, over 800 plants might have promising potential against DM complications [17]. Only a few plants used in conventional medicine have been phytochemically, biologically or clinically tested [18,19]. Importantly, herbal medicine also has side effects, often overlooked by the patients [20]. Therefore, more clinical research needs to help patients safely use plants.

Herbal remedies have been traditionally used to maintain general health and well-being. Given the high prevalence of DM in the population of the Caribbean basin, there is popular knowledge on medicinal plants to prevent or treat DM [21,22,23]. This traditional medicine takes advantage of the extraordinary biodiversity of the Caribbean islands, considered a global biodiversity hotspot [24,25]. Furthermore, food technology research is helping the scientific community to decipher new valuable information regarding the nutritional composition and the antidiabetic potential of edible plants and fruits [26,27]. In particular, this review focuses on the description of antidiabetic plants from the Caribbean basin, exploring their pharmacological mechanisms to propose the Caribbean flora as a source of innovative bioactive natural products to treat and prevent DM and associated complications. Exploring the anti-DM bibliography, we have selected 7 Caribbean plants, 5 indigenous and 2 exotic species, traditionally used in Caribbean folk medicine. In vivo and in vitro preclinical, experimental evidence of hypoglycemic influence are available (Table 1 and Table 2). These seven species are *Anacardium occidentale* L. (Anacardiaceae), *Hyptis suaveolens* (L.) Poit. (Lamiaceae), *Persea americana* Mill. (Lauraceae), *Psidium guajava* L. (Myrtaceae), *Momordica charantia* L. (Cucurbitaceae), *Tecoma stans* (L.) Juss. ex Kunth (Bignoniaceae) and *Phyllanthus niruri* L. (Phyllanthaceae). We classified these plants regarding their pharmacological activity and the mechanism of action of their key active phytocomponents.

## 2. The Caribbean Flora: A Unique and Diverse Source of Pharmacological Compounds

Many natural products isolated from plants, including polypeptides, carotenoids, flavonoids, alkaloids, terpenoids, saponins, tannins and glycosides, have previously shown antidiabetic properties [72,73]. The antidiabetic acting mechanisms of the phytochemicals found in plants can be categorized into six groups regarding their pharmacological mechanism: (I) glucose metabolism modulators, (II) hypolipidemic effectors, (III) pancreatic effectors, (IV) antioxidative effectors, (V) diabetic-related complications modulators and (VI) insulin-mimetic and insulin-sensitizer modulators [74].

The first group contains phytochemicals that modulate metabolic pathways in which glucose acts as a substrate or as a product. Therefore, they affect gluconeogenesis, glycogenolysis, pentose phosphate pathways and glycogenesis. This group is also comprised of phytochemicals that affect glucose uptake and compounds that show α-glucosidase and α-amylase inhibitory activity. Group II harbors phytochemicals that lower triglyceride levels and cholesterol content, impacting hyperlipidemia, a pathophysiological feature of DM [75]. Group III is comprised of bioactive compounds that can protect against β cell damage, increase their proliferation and stimulate insulin secretion. On the other hand, groups IV and V include phytochemicals that protect from DM-derived oxidative stress and DM-related complications. Finally, natural products that mimic and enhance insulin activity can also be found in herbal extracts (group VI). Interestingly, phytochemicals from plants can display multiple anti-DM effects. An illustrative example is a case of flavonoids that can be categorized into several groups (at least I and III) [76]. 

The Caribbean flora constitutes an untapped reservoir of natural biodiversity and a vast source of natural products. It has evolved under conditions of extreme environmental stress. It is exposed to multiple stressors, including high levels of UV radiation, high temperatures, anaerobic soils, strong winds, high salt and sulfide concentrations, oxidative stress, nutrient deficiency, extreme seasonality, phytopathogens and herbivory. The ability of Caribbean species to grow under these conditions is mainly due to specialized physiological processes that affect the chemical responses of the plants, triggering the biosynthesis of unique metabolites that include hydrocarbons, terpenoids, polyphenols, alkaloids, flavonoids, and quinones [77]. Several species were cited on different ethnopharmacological surveys developed by the TRAMIL network (Program of Applied Research to Popular Medicine in the Caribbean, www.tramil.net) (accessed on 28 September 2021) [78]. This multidisciplinary project aims to validate scientifically the pharmacological potential of medicinal plants used in primary care in multiple regions of the Caribbean basin. This multidisciplinary network created in the 1980s has dev eloped a research program to valorize and make available traditional knowledge of the uses of medicinal Caribbean flora and practical and affordable treatments that harmonize with the popular traditions of the Caribbean basin. The main objective of TRAMIL is to assure access to practical ethnopharmacological knowledge of the local flora, validated previously by scientific methods, for the local people, medical and paramedical staff and the academy or institutions related to environmental health conservation. Additionally, another purpose of the program is to highlight popular herbal medicine as a complementary resource to allopathic medicine and fight for the value of the Caribbean culture and ancestral traditions. 

**Figure 1:**
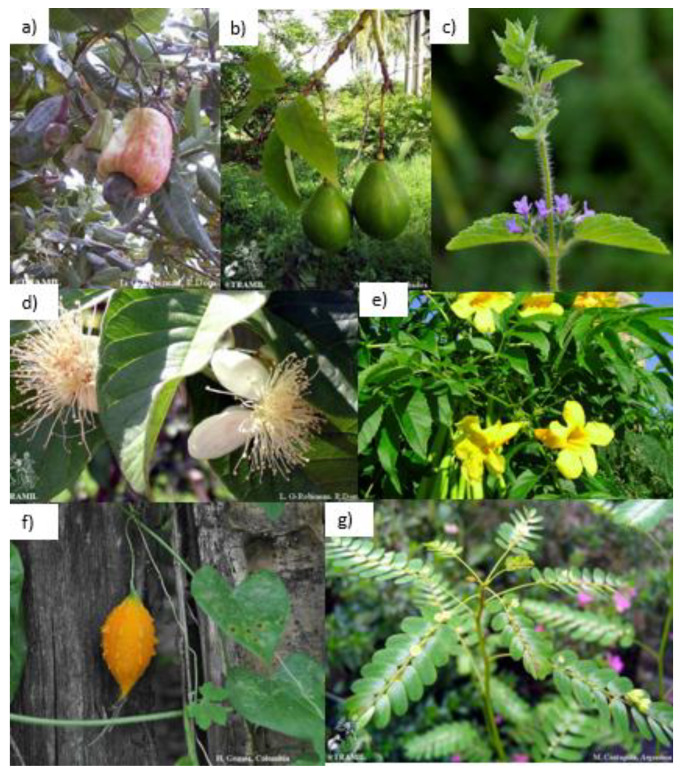
Representative picture of medicinal Caribbean flora discussed through the review. (**a**) *Anacardium occidentale* L., (**b**) *Hyptis suaveolens* (L.) Poit., (**c**) *Persea americana* Mill., (**d**) *Psidium guajava* L., (**e**) *Tecoma stans* (L.) Juss. ex Kunth, (**f**) *Momordica charantia* L. and (**g**) *Phyllanthus niruri* L. (source: www.tramil.net).

**Figure 2:**
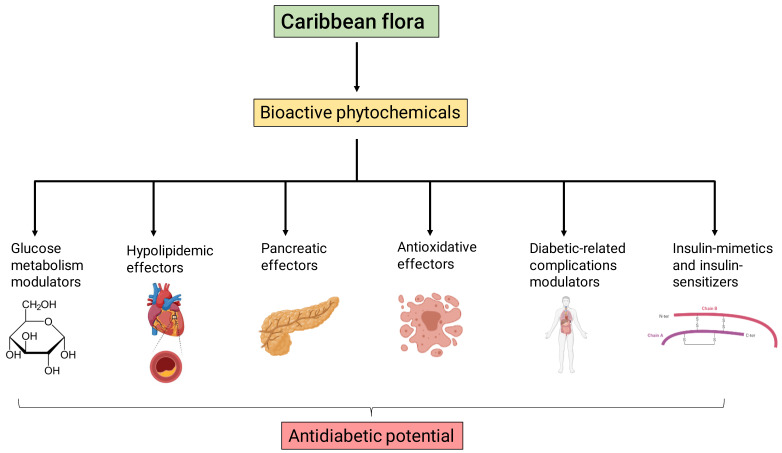
Hypothetical model of antidiabetic action mechanisms of bioactive phytochemicals from the Caribbean flora. Phytochemical compounds may act as glucose metabolism modulators, hypolipidemic effectors, pancreatic effectors, anti-oxidative effectors, diabetic-related complication modulators and insulin mimetics/sensitizers.

Here we summarize the available literature about the antidiabetic potential of these Caribbean species. We compile in vitro and in vivo experimental evidence supporting a beneficial impact of extracts or isolated fractions that follow the above-described antidiabetic action mechanisms (Figure 1 and Figure 2).

### 2.1. Anacardium occidentale L.

**Figure 3:**
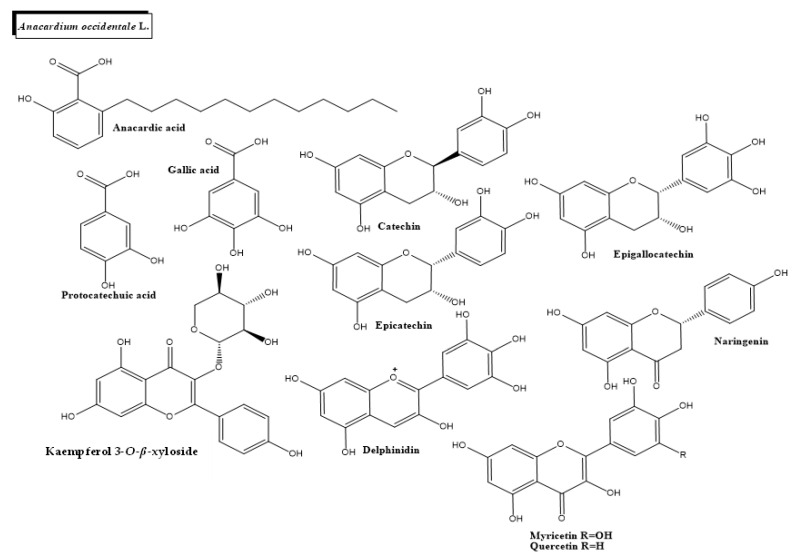
Chemical structures of bioactive phytocompounds with reported antidiabetic properties identified in *Anacardium occidentale* L.

*A. occidentale* L. (Figure 1a and Figure 3) is a tropical tree widely used for medicinal and nutraceutical purposes. Leaf, seed, and bark extracts from *A. occidentale* L. have been shown to have antidiabetic, antibacterial, anti-inflammatory, and antiulcerogenic properties. Ethanolic extracts of *A. occidentale* stem bark and flowers contain flavones, phenolic compounds, triterpenes, xanthones, anacardic acids (AA) (e.g., 6-pentadecyl salicylic acid) and gallic acid [79]. The antidiabetic potential of *A. occidentale* is also being explored using computational approaches. According to a ligand-based prediction model, 8 different hit compounds from the plant (kaempferol 3-*O-β*-D-Xyloside, myricetin, quercetin-3-*O- β*-D-arabinofuranoside, delphinidin, gallic acid, quercetin-3-*O*-D-galactopyranoside, (+) catechin, protocatechuic acid, epigallocatechin, naringenin and (−) epicatechin) have potential action against glutamine-fructose-6-phosphate aminotransferase 1 (GFAT1) and dipeptidyl peptidase-4 (DPP-4), two promising therapeutic targets for DM management [80].

Several in vivo studies strengthen the antidiabetic potential of plant extracts of *A. occidentale* [40,41,81,82]. In mice fed with a high fat and high sucrose diet, AA therapy significantly decreased serum insulin and the homeostatic model assessment of insulin resistance (HOMA-IR) levels, resulting in slightly lower liver weights and significantly lower levels of liver fats, total cholesterol, and low-density lipoprotein cholesterol [28]. The methanolic extract (100 mg/kg) of *A. occidentale* leaves had major beneficial effects in lowering blood glucose levels in streptozotocin-induced diabetic rats. The impact of *A. occidentale* extracts was similar to pioglitazone, a standard medicine used for DM management. After administration of 100 mg/kg of plant extract, the rats’ blood glucose levels showed an 8.01% and 19.25% decrease in their fasting blood glucose levels after 15 and 30 days, respectively [40]. Another study reported that the aqueous extract (175 mg/kg) of *A. occidentale* leaves showed protection against the hyperglycemic effects of streptozotocin in rats [41].

The role of AA as a therapeutic agent on metabolic disorders, including fatty liver disease and DM, has been deeply investigated. AA in vitro administration reduced lipid accumulation in 3T3-L1 cells without observed cytotoxicity. Adipocyte differentiation was inhibited by reducing the expression of the fatty acid synthase (FAS) and peroxisome proliferator-activated receptor-gamma (PPAR-γ), two adipogenesis-related markers [28]. Hydroethanolic extracts of *A. occidentale* seeds and their active ingredient, AA, induced glucose absorption into C2C12 muscle cells in a dose-dependent manner. Nonetheless, the extracts from other plant sections (leaves, bark, and apple) were inactive [29]. In addition to AA, it has been suggested that other phytocompounds of the plant extract could be responsible for its antidiabetic effect. For example, terpenoids and coumarins or stigmast-4-en-3-ol and stigmast-4-en-3-one, two steroidal compounds found in bark extracts, might have antidiabetic properties [42,43].

Compelling literature supports that, in addition to its hypoglycemic and hypolipidemic impact, *A. occidentale* might affect different antidiabetic acting mechanisms. For example, the oral administration of the ethanolic extract of *A. occidentale* flowers revealed anti-inflammatory behavior in diabetic mice with sepsis and extended mice lifespan. The flower extract of the plant stimulated the recruitment and proliferation of macrophages and neutrophils and decreased IL-6, MCP-1, and TNF-α, three immunomodulatory cytokines [83]. In a mouse model of colitis, the oral administration of cashew nuts from *A. occidentale* showed anti-inflammatory and antioxidant properties, reducing neutrophil infiltration and colon damage and lowering malondialdehyde (MDA) and pro-inflammatory cytokine levels. Additionally, cashew nut administration enhanced manganese superoxide dismutase (MnSOD) antioxidant activity and inhibited NF-kβ nuclear factor, a key player during inflammation [44]. *A. occidentale* bark extract has also been reported to stimulate pancreatic β cell islet cells. The plant extract acted on β-cells similarly to sulfonylurea drugs, stimulating insulin discharge in a glucose-dependent manner [82].

Despite many in vitro and in vivo studies using *A. occidentale* extracts, the information about its toxicity and safety is scant. Regarding the acute toxicity of the hexane leaf extract on mice, the LD_50_ was reported to be 16 g/kg. Additionally, the oral subchronic treatment during 56 days at doses of 10 and 14 g/kg suggests liver and kidney toxicity since urea, creatinine and transaminase levels are impaired [84]. 

Furthermore, recent findings suggest that other plants from the *Anacardiun* genus can also be used in DM management. For instance, the ethanolic extract of *Anacardium humile* St. Hil leaves showed important in vitro antiglycation and antioxidant activity. It inhibited α-amylase activity in RAW264.7 cells, a mouse macrophage cell line [85]. 

### 2.2. Hyptis suaveolens (L.) Poit.

**Figure 4:**
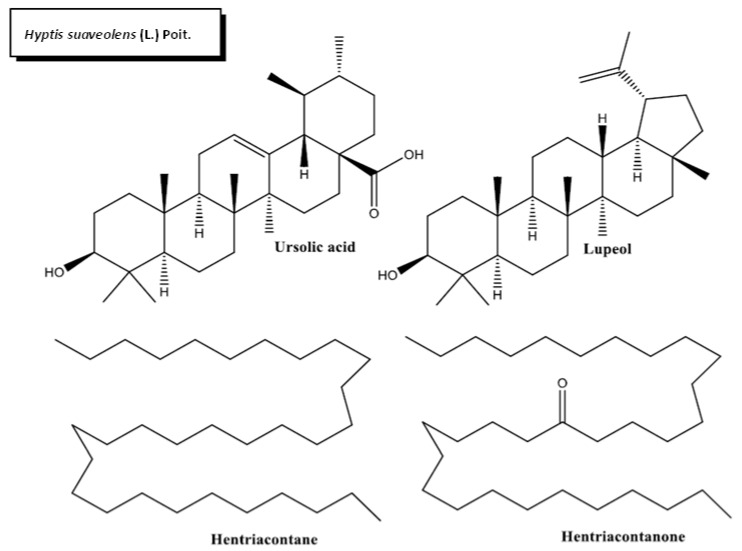
Chemical structures of bioactive phytocompounds with reported antidiabetic properties identified in *Hyptis suaveolens* (L.) Poit.

*H*. *suaveolens* (L.) Poit. (Figure 1b and Figure 4) is an aromatic medicinal plant commonly used in Central and South America and the West Indies. Due to its pharmacological potential, it has been used for multiple therapeutic purposes. Evidence suggests that *H. suaveolens* possesses anti-cancerous, antibacterial, antifungal, and antihyperglycemic activity [86].

*H. suaveolens’* phytochemical profile contains alkaloids, carbohydrates, flavonoids, tannins, steroids, and terpenes [86]. Other phytochemical constituents present in *H. suaveolens* leaves are hentriacontane, hentriacontanone, lupeol, diterpenoids, and triterpenoids. There is scant information about the pharmacological potential of these phytocompounds of *H. suaveolens.* The major therapeutical activity of the plant might reside in ursolic acid (UA), a pentacyclic triterpenoid. UA is a potent hypoglycemic agent acting as an insulin secretagogue and insulinomimetic. It enhances insulin secretion, transportation and uptake by the glucose transporter protein (GLU4) by stimulating the intracellular accumulation of calcium [45,87].

Growing evidence informs the promising potential of *H. suaveolens* extracts for DM management. The oral administration of the ethanolic extract of the leaves had a hypoglycemic impact in diabetic rats, also reducing cholesterol and triglyceride levels [46]. Additionally, the plant’s aqueous ethanolic and petroleum ether extracts had comparable benefits to standard insulin as they significantly reduced blood glucose levels by stimulating peripheral glucose utilization [30]. The chloroform fraction of *H. suaveolens* had antioxidant potential in different diabetic rat models and inhibitory potential on salivary α-amylase [88]. These findings regarding *H. suaveolens* as an antihyperglycemic agent and for promoting the improvement of oxidative stress present this Caribbean species as a promising source of phytocompounds to fight against DM. Unfortunately, there is no information regarding *H. suaveolens’* side effects and toxicity. Further studies are needed to address its suitability for pharmaceutical purposes.

The antidiabetic potential of other plant species belonging to the *Hyptis* genus has also been tested. In streptozotocin-induced diabetic rats, the administration of the ethanolic extract of *Hyptis verticillata* jacq. leaves decreased HbA1c and fasting blood glucose levels [89]. Analogously, the ethanolic extract (250 mg/kg and 500 mg/kg) of *H. verticillata* ameliorated dyslipidemia (increasing the cardioprotective index and lowering the atherogenic coefficient and the atherogenic and coronary risk indices) [90]. The *H. verticillata* extract diminished oxidative stress (decreasing MDA and stimulating catalase, glutathione peroxidase and superoxide dismutase (SOD)) and also ameliorated hepato-renal damage and lowered blood glucose levels of streptozotocin-induced rats [90].

### 2.3. Persea americana Mill.

**Figure 5:**
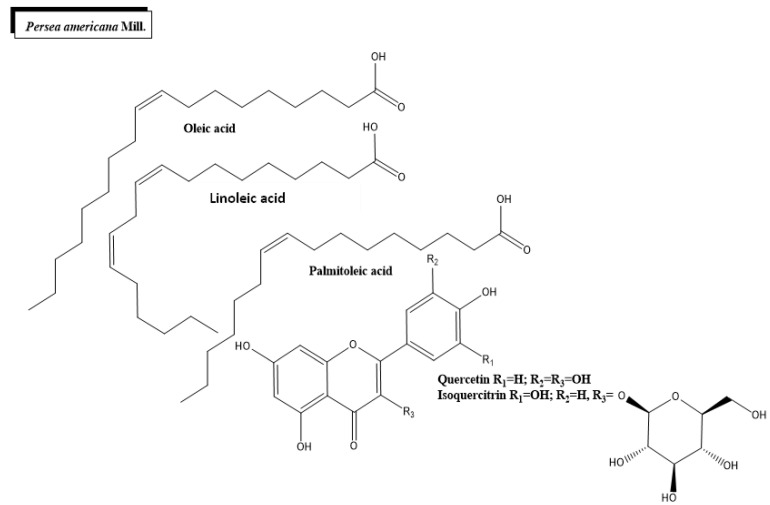
Chemical structures of bioactive phytocompounds with reported antidiabetic properties identified in *Persea americana* Mill.

Persea americana Mill. (Figure 1c and Figure 5) is an arboreal plant originally from Central and South America and is known for its fruit, commonly referred to as avocado. High levels of bioactive compounds have been described in *P*. *americana*, including phenol compounds, organic acids, alkaloids, diterpenoids and amino acids [91]. Avocado fruit has many phytonutrients, including oleic acid, unsaturated fatty acids, palmitoleic, linoleic acid, acetogenins and carotenoids [92]. The nutraceutical richness of avocado is well known, and several metabolites of the fruit have anti-inflammatory, anticancer, antimicrobial and cardioprotective properties [93]. Among these compounds, two glycosylated flavonoids (isoquercitrin and quercetin) have a hypoglycemic effect. These molecules act by inhibiting GLUT2 (a bidirectional transmembrane passive glucose transporter) or facilitating GLUT4 (insulin-sensitive glucose transporter that facilitates glucose uptake by cells) membrane translocation [94,95,96].

*P*. *americana* leaves have been proposed as an interesting phytopharmaceutical source. In a comparative study, the effects of aqueous, ethanolic and methanolic leaf extracts of the plant were assessed in nicotinamide and streptozotocin-induced diabetic rats. The methanolic extract displayed the highest antidiabetic potential, but all the extracts significantly reduced glucose blood levels and ameliorated the hyperlipidemic state of the diabetic rats [47]. The authors also suggested that the hydroethanolic extract might stimulate the regeneration of the islets of Langerhans from the pancreas. In another study, the hydroalcoholic extract of *P. americana* leaves reduced blood glucose content in diabetic rats, restoring the intracellular energetic balance through the phosphorylation and the activation of protein kinase B (PKB), a central player in the AKT/PKB signaling pathway [48]. This signal transduction pathway is activated in response to insulin or growth factors and mediates multiple cell responses such as nutrient metabolism, proliferation, cell growth and apoptosis [97]. The activation of PKB promotes glyconeogenesis through glycogen synthase activation [98]. Nevertheless, since *P. americana* leaves can modify normal renal function [99], further studies regarding their safety are needed.

*P. americana* seeds have also demonstrated antidiabetic effects. The oral administration of an aqueous extract of *P*. *americana* seeds to alloxan-induced diabetic rats had significant tissue-protective effects on the pancreas, liver, and kidney and lowered blood glucose levels. The hypoglycemic effect was similar to glibenclamide, a drug used for T2D management [49]. 

Additionally, *P. americana* extracts have been linked to the inhibition of T2D key enzymes. The phenolic extracts of *P. americana* leaves and fruit inhibit both α-amylase and α-glucosidase in a dose-dependent manner [50]. According to its IC_50_ value (half-maximal inhibitory concentration), the peel had the highest inhibitory activity for α-amylase, while the leaves had the most inhibitory action for α-glucosidase. Moreover, the phenolic extracts inhibited induced-lipid peroxidation in the pancreas in a dose-dependent manner [50].

Apart from *P. americana*, other plants from the *Persea* genus have shown promising antiglycation and antidiabetic properties. In alloxan-induced diabetic rabbits, 24 days of treatment with the crude extract of *Persea duthieion* reduced the animals’ body weight and had significant antihyperglycemic effects. In particular, the ethyl acetate fraction of the extract had the highest antidiabetic activity [100]. Moreover, the methanolic leaf extract of *Persea indica* inhibited BSA glycation in vitro and inhibited α-amylase, α-glucosidase, and aldose reductase activity. The antiglycative action was higher than that observed for aminoguanidine, a glycation protector compound [101].

### 2.4. Psidium guajava L.

**Figure 6:**
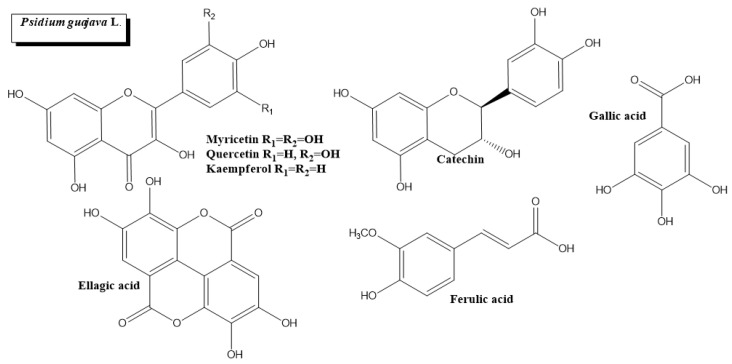
Chemical structures of bioactive phytocompounds with reported antidiabetic properties identified in *Psidium guajava* L.

*Psidium guajava* L. (Figure 1d and Figure 6) is a medicinal tree native to South America, Central America and the Caribbean. Almost every part of *P. guajava*, including its fruits, leaves, bark, and roots, has been widely used to treat or prevent DM and protein glycation and a variety of affections such as gastrointestinal disorders, cancer, inflammation, diarrhea, and hypertension [102,103].

*P. guajava* leaf extract contains anthraquinones and ellagic acid, two active compounds with inhibitory activities for α-amylase, tyrosinase and hyaluronidase [104]. Significant in vitro evidence supports the antidiabetic properties of *P. guajava*. Quercetin, a flavonoid found in the leaf’s aqueous extract, improved glucose absorption in cultured rat hepatocytes [105]. The ethanolic extract of *P. guajava* leaves and bark stimulated glucose uptake in murine C2C12 skeletal muscle cells and enhanced triglyceride accumulation in 3T3-L1 adipocyte-like cells [31].

The leaves of *P. guajava* have a high tannin content with a non-specific inhibitor activity of digestive enzymes, including trypsin, α-amylase, lipase and α-glucosidase. Flavonols from *P. guajava* leaves, such as myricetin, quercetin, and kaempferol, cause inhibition of maltase, α-amylase and sucrose [32,33]. Furthermore, flavonol glycosides from *P. guajava* have inhibition activity on dipeptidyl peptidase IV, a major player in glucose metabolism [106]. 

*P. guajava* has hypoglycemic consequences in many in vivo studies. The water extract of *P. guajava.* leaves lowered fasting plasma glucose, total cholesterol, low-density lipoprotein cholesterol, and glycosylated serum protein levels in vitro, reducing hyperglycemia and hyperlipidemia [51]. The oral administration of leaf *P. guajava* extracts reduced fasting blood glucose levels and increased glucose-6-phosphatase dehydrogenase, glycogen, hexokinase and insulin levels in diabetic-induced rats [52]. The authors propose that the PI3K/AKT pathway plays a major role in this hypoglycemic effect since AMPK, p-AMPK, AKT, p-AKT, IRS-1, IRS-2, PI3K and GLUT2 are activated. Another study reported that the long-term administration of aqueous and ethanolic soluble solid extracts of *P. guajava* leaves to diabetic rats improved glucose utilization and increased insulin plasma levels [53]. Interestingly, increased hepatic hexokinase, phosphofructokinase and glucose-6-phosphate dehydrogenase activity were detected in diabetic rats that were administered the aqueous extract. In contrast, in rats treated with ethanolic extract, the increment was observed only in hepatic hexokinase and glucose-6-phosphate dehydrogenase. In alignment with these results, the intraperitoneal injection of *P. guajava* extract had an antihyperglycemic activity mediated through the protein tyrosine phosphatase 1B (PTP1B), a negative regulator of the leptin and insulin signaling pathways [54]. Given that PTP1B has been proposed as a promising therapeutical target for DM and cancer [107], *P. guajava* arises as a valuable phytopharmaceutical agent. In addition, the isolated polysaccharide fraction of *P. guajava* leaves lowered the total triglyceride and cholesterol contents, decreased fasting glucose levels and enhanced the superoxide dismutase activity in streptozotocin-induced diabetic rats [55]. 

*P. guajava* leaf aqueous extract also shows a strong inhibitory effect on Amadori products (fructosamines) that are intermediates in the production of AGEs. Gallic acid, catechin and quercetin are phenolic compounds from the leaves believed to possess antiglycation properties [34]. Furthermore, ferulic acid, a phytochemical present in *P. guajava* leaf extract, shows an important antiglycative and anticoagulant activity restoring the antithrombin III function previously inhibited by methylglyoxal, a potent precursor of glycation [35]. 

*P. guajava* has also been proposed as a therapeutic agent against DM-associated complications. The plant leaf extracts’ ethyl acetate fraction significantly reduced serum fructosamine and HbA1c levels. They lowered the expression of transforming growth factor β (TGF-β1), connective tissue growth factor (CTGF) and B-type natriuretic peptide (BNP) in the myocardium of diabetic rats, suggesting a cardioprotective role for the *P. guajava* extract [56]. The intraperitoneal administration of *P. guajava* triterpenoids resulted in increased serum insulin levels and insulin sensitivity index and ameliorated the renal damage of streptozotocin-induced diabetic rats [57]. These results highlight the nephroprotective potential of the plant. However, further studies are needed to evaluate the toxicity of *P. guajava* extracts since there is no information available in the literature.

### 2.5. Tecoma stans (L.) Juss. ex Kunth

**Figure 7:**
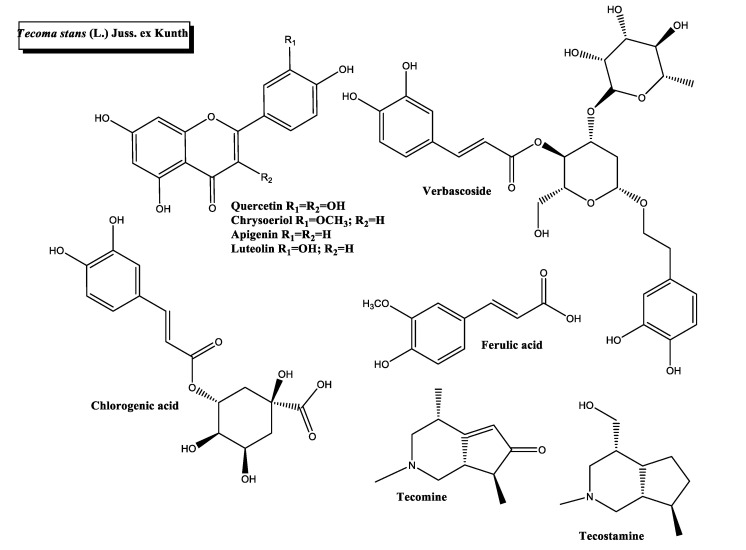
Chemical structures of bioactive phytocompounds with reported antidiabetic properties identified in *Tecoma stans* (L.) Juss. ex Kunth.

*Tecoma stans* (L.) Juss. ex Kunth (Figure 1e and Figure 7) is a flowering tree native to Central and South America. It is one of the most widely used medicinal plants for diabetes management in several countries, including the USA and Mexico [58]. Traditionally, *T. stans* extracts have been used for antioxidant, analgesic, hepatoprotective, antibacterial and antiplasmodic purposes [108,109].

Approximately 120 different compounds have been isolated from *Tecoma stans* (L.) Juss. ex Kunth, including terpenoids, flavonoids, glycosides, unsaturated fatty acids and carotenoids [110]. *T. stans* produce elevated levels of phytochemical substances with pancreatic lipase inhibitory activity and, thus, antidiabetic potential. Chrysoeriol, apigenin, luteolin, and verbascoside from *T. stans* leaves showed lipase inhibitory activity, with chrysoeriol and apigenin being the most bioactive compounds [36]. Interestingly, these two phytochemicals have been linked to DM management. Chrysoeriol is a methoxyflavone that acts as an α-amylase enzyme inhibitor, and apigenin is a flavonoid with antidiabetic potential, lowering glucose blood levels, malonaldialdehyde content and insulin resistance index [59,60,111]. In addition, chlorogenic acid, tecostatine and tecomine are three phytochemicals from *T. stans* thought to have a hypoglycemic effect. Chlorogenic acid reduced postprandial peaks of glucose and decreased liver triacylglycerol levels and fasting cholesterol plasma content in obese, hyperlipidemic and insulin-resistant rats [61]. Tecomine has an in vitro hypoglycemic impact, promoting a significant stimulation of the basal uptake rate of glucose in normoglycemic rat adipocytes [112]. Tecostatine and tecostatine were proposed as hypoglycemic agents more than 70 years ago [113], although the antidiabetic effects of these compounds remain controversial. 

In vivo evidence supports a beneficial effect of *T. stans* extracts in a diabetic context [58]. These exert at least four anti-diabetic properties functions, including intestinal α-glucosidase suppression, postprandial antihyperglycemic effect, hypocholesterolemic, and hypotriglyceridemic effect. Additionally, the leaf extract of *T. stans* stimulated the glucose uptake in insulin-sensitive and insulin-resistant murine or human adipocytes [37]. 

Recent studies report that the antidiabetic potential of *T. stans* is being examined in clinical trials. The effectiveness of an herbal mixture made of *Guazuma ulmifolia* and *T. stans* was assessed in a randomized, double-blind, placebo-controlled trial. The group of T2D patients that were administered with the mixture showed a significant decrease in waist circumference, fasting glucose levels and HbA1c content [62]. Furthermore, the safety and toxicity of *T. stans* is a relevant issue that needs to be evaluated. The subchronic treatment with the plant extract showed no mortality and no adverse effects in rats [114]. 

### 2.6. Momordica charantia L.

**Figure 8:**
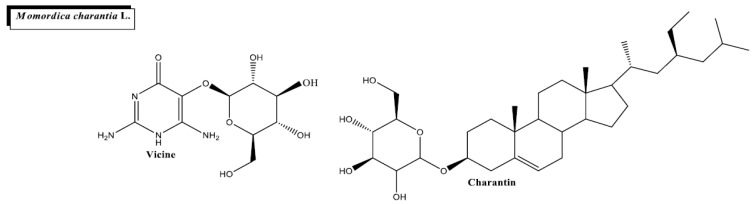
Chemical structures of bioactive phytocompounds with reported antidiabetic properties identified in *Momordica charantia* L.

*Momordica charantia* L. (Figure 1f and Figure 8) is a health-promoting tropical plant grown in the Caribbean region, Africa and Asia. Since every part of the plant possesses therapeutical effects, it has been widely used in traditional medicine. Nevertheless, with its edible fruit, the bitter melon is one of the most-used parts for pharmacological purposes. Multiple reports show *M. charantia*’s antiobesity, antimicrobial, anti-inflammatory and anticancer properties [115,116,117].

The high richness of phytochemicals present in *M*. *charantia* makes this plant suitable for use in DM and DM-related complication management. *M. charantia* contains anti-diabetic compounds with anti-α-glucosidase activity such as charantin, insulin-mimetic metabolites such as the polypeptide-p and insulin-sensitizing chemicals such as triterpenoids [118]. *M. charantia* also has alkaloids such as vicine, which can stimulate the uptake of cellular glucose and reduce insulin resistance [68]. Other chemicals with antidiabetic activity found in *M. charantia* include cucurbutanoid compounds, sterol glycosides and flavonoids [119]. The active components from *M. charantia* have 3 different antidiabetic mechanisms of action (as reviewed in [32]): (I) affecting the activity of glucose metabolism enzymes (enhancing glycogen synthase, glucose-6-phosphatase and glucose-6-phosphate dehydrogenase), (II) stimulating β cells from the pancreas and thus increasing insulin production and (III) serving as ligands of peroxisomal proliferator-activated receptors (PPARs), a family of receptors that regulate fat and glucose metabolism.

The hypoglycemic therapeutical effect of *M. charantia* has been tested in numerous in vivo and in vitro studies. The fruit extract showed significant antihyperglycemic activity by reducing the percentage of HbA1c and blood glucose levels and by inhibiting glycogenolysis in vitro [63]. Similarly, the oral administration of the aqueous extract of the whole fruit reduced fasting blood glucose levels in diabetic rats. It showed comparable effects to glibenclamide, a diabetic synthetic drug [64]. Additionally, the glucose tolerance curve was altered after the administration of the plant extract in several studies [65,66,67,68,69,70,71,72,73,74,75,76,77,78,79,80,81,82,83,84,85,86,87,88,89,90,91,92,93,94,95,96,97,98,99,100,101,102,103,104,105,106,107,108,109,110]. 

The probiotic-rich fermented extract of *M. charantia* has been proposed as a promising complementary agent for diabetes with antioxidant properties. *M. charantia* juice was fermented with *Lactobacillus fermentum* LLB3, a lactic acid bacterium, increasing its antioxidant properties [66]. In this study, streptozotocin-induced diabetic rats were fed with fermented *M. charantia* juice, non-fermented M. *charantia* juice, or administered acarbose, a typical anti-diabetic drug. The fermented juice showed more anti-diabetic properties than the non-fermented one. Rats fed with fermented juice and with non-fermented juice reported a significant reduction in fasting blood glucose levels (109.1 ± 2.07 mg/mL and 164.3 ± 2.21 mg/mL, respectively) in postprandial blood glucose levels (120.1 ± 2.49 mg/mL and 174.9 ± 2.04, respectively) mg/mL and an increase in SOD levels (61.8 ± 4.98 and 50.1 ± 5.68, respectively) when compared with diabetic rats that were given only water. However, the antidiabetic effects were lower than those observed using acarbose. Rats within this group reported the lowest fasting and postprandial blood glucose levels (88.9 ± 2.16 mg/mL and 100.4 ± 2.90 mg/mL, respectively) and the highest SOD levels (84.3 ± 3.95). In another study, the aqueous extract of the bitter melon significantly decreased blood glucose levels and HbA1c content and increased tissue glycogen serum insulin and glucagon-like peptide 1 (GLP-1) in streptozotocin-induced diabetic rats [67]. Since GLP-1 enhances insulin secretion, upregulation of GLP-1 has beneficial antidiabetic effects. In fact, GLP-1 receptor agonists are used for T2D management [120]. Furthermore, 8-week treatment using a dose of 400 mg/kg of an ethanolic *M. charantia* extract to streptozotocin-induced diabetic rats resulted in lowered serum glucose and in a reduction of the suppressor of cytokine signaling-3 (SOCS-3), c-Jun N-terminal kinase (JNK) and GLUT-4 content [121], two key players that modulate insulin resistance [122,123]. Despite the wide use of bitter melon in cuisine, it would be beneficial to characterize its use better for therapeutical purposes to prevent toxicity or possible side effects.

*M. charantia* can also help to prevent complications from kidney failure. A recent study showed that *M. charantia* extracts improved blood glucose levels, defended against diabetic nephropathy, decreased body weight loss, and preserved hyperglycemia in rats with streptozotocin-induced T2D [69].

### 2.7. Phyllanthus niruri L.

**Figure 9:**
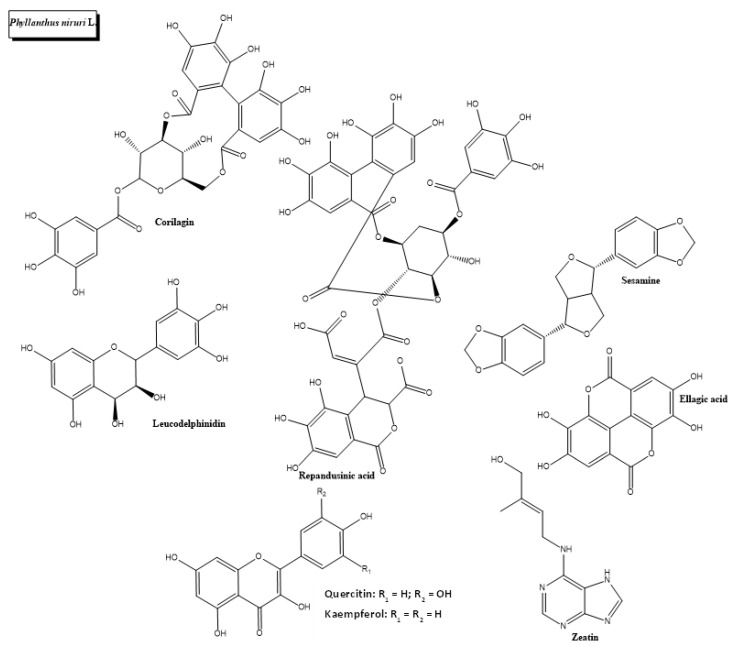
Chemical structures of bioactive phytocompounds with reported antidiabetic properties identified in *Phyllanthus niruri* L.

*Phyllanthus niruri* L. (Figure 1g and Figure 9) is a perennial tropical small herb found in the coastal areas of India, in the Caribbean basin, Brasil and the Amazon rainforests. Many bioactive compounds have been identified in *P. niruri* with multiple pharmacological properties. Phytochemical research shows that the leaf extract contains alkaloids, flavonoids, saponins, tannins, lignins, terpenoids and coumarins but not hormones, glycosides or resins [124]. Due to its bioactive constituent diversity, the aerial parts of the plant have been used worldwide in traditional medicine. 

Several studies highlight the anticholesterolemic, anti-inflammatory, anti-hyperuricaemic and potent anticancer and antimicrobial properties of *P. niruri* [125,126,127].

P. niruri has been tested as a hypoglycemic agent regarding its antidiabetic potential. The administration of the plant extract improved the lipid profile and lowered the serum glucose levels of T2D rats [70]. The authors of this study claimed that *P. niruri* protects against diabetes-related renal disorder and pointed out that this herb may be used to treat diabetic nephropathy. Another study reported that oral administration of *P. niruri* extract from its aerial parts to alloxan-induced diabetic rats lowered HbA1c, decreased blood glucose levels and increased liver glycogen amount [71].

Bioactive phytocompounds of P. niruri also enhance other antidiabetic acting mechanisms. For example, the α-glucosidase inhibitors corilagin and repandusinic acid A also have been discovered in the water extracts of *P. niruri* [38]. Additionally, the aqueous extract of the leaves was reported to have antioxidant activity and prevents AGE formation in diabetic rats [39]. The *P. niruri* extract inhibited superoxide and hydroxyl radical formation and possessed hydrogen peroxide scavenging activity by ameliorating glutathione peroxidase, catalase and superoxide dismutase function and lowering MDA and lipid peroxidation products. Unfortunately, there is no available information about *P. niruri*’s possible harmful effects. Further studies should be implemented to address the plant toxicity and dosage.

In the *Phyllanthus* genus, several other plant species have shown antidiabetic potential. An in silico study revealed that ellagic acid, phytoestrogens, sesamine, kaempferol, zeatin, quercetin, and leucodelphinidin, natural products that can be found in *Phyllanthus emblica* L. (Figure 9), possess antidiabetic activity [128]. Moreover, *Phyllanthus amarus* has reported diuretic, hypoglycemic and hypotensive effects in humans [129]. Finally, *Phyllanthus acidus*. Skeels has also shown antioxidant, hypoglycemic and α-glucosidase inhibitory activity [130]. 

## 3. Conclusions and Further Perspectives

DM is a recurrent condition that calls for ongoing and long-term treatment and possesses a challenge for worldwide healthcare. In this scenario, natural products from plants represent an abundant pharmacological source to treat or prevent DM and its associated complications. A significant proportion of diabetic patients are regular users of these traditional medicines. Given that the Caribbean Basin represents a unique ethnopharmacological relevant area due to its floral diversity, the Caribbean flora represents an opportunity to discover novel bioactive phytocompounds to combat DM. This review describes the antidiabetic potential of seven widely used plants in the Caribbean region in folk medicine. However, the pharmacological information for each plant is scant. Further investigation is required to identify novel phytochemicals and determine which molecular and cellular pathways are involved in the protective anti-diabetic effect of these traditional herbal medicines. Additionally, a more intense effort in research is imperative to define the effective dosage and toxicity of the various plant extracts that are commonly used.

## Figures and Tables

**Table 1:** General overview of plants from the Caribbean flora described in this review in which in vitro studies are available. Information about the origin, part of the plant used, type of extract, antidiabetic activity and references are provided.

Plant	Origin	Part Used	Extract	Activity	References
*Anacardium occidentale* L. (Anacardiaceae)	Indigenous	Cashew seed Cashew nuts	Hydroethanolic	Hypolipidemic	[28,29]
*Hyptis suaveolens* (L.) Poit. (Lamiaceae)	Indigenous	Aerial parts	Ethanolic Aqueous-ethanolic Petroleum ether Chloroform fraction	Hypoglycemic	[30]
*P. guajava* L. (Myrtaceae)	Indigenous	Leaves Bark	Aqueous	Hypoglycemic α-amylase inhibitor α-glucosidase inhibitor Hypolipidemic Antiglycation	[31,32,33,34,35]
*Tecoma stans* (L.) Juss. ex Kunth (Bignoniaceae)	Indigenous	Leaves	Hydroalcoholic Aqueous	Antihyperlipidemic	[36,37]
*Phyllanthus niruri* L. (Phyllanthaceae)	Exotic	Leaves	Ethanolic Aqueous	Antihyperlipidemic Antihyperglycemic α-glucosidase inhibitor Antioxidant	[38,39]

**Table 2:** General overview of plants from the Caribbean flora described in this review in which in vivo studies are available. Information about the origin, part of the plant used, type of extract, antidiabetic activity and references are provided.

Plant	Origin	Part Used	Extract	Activity	References
*Anacardium occidentale* L. (Anacardiaceae)	Indigenous	Leaves Bark Cashew nuts	Hexane Aqueous Methanolic Ethanolic	Hypoglycemic Hypolipidemic Anti-inflammatory antioxidant	[39,40,41,42,43,44]
*Hyptissuaveolens* (L.) Poit. (Lamiaceae)	Indigenous	Leaves	Ethanolic Aqueous-ethanolic	Insulin-mimetism Insulin secretagogue Hypoglycemic hypolipidemic	[45,46]
*Persea americana* Mill (Lauraceae)	Indigenous	Leaves Fruit Seeds	Hydroalcoholic Phenolic Aqueous Ethanolic Methanolic	Hypoglycemic α-amylase inhibitor α-glucosidase inhibitor DM-associated complication protection (nephroprotection and hepatoprotection) Hypolipidemic Pancreatic protector	[47,48,49,50]
*P. guajava* L. (Myrtaceae)	Indigenous	Leaves	Aqueous Ethanolic	Hypoglycemic Hypolipidemic Antiglycation DM-associated complication protection (cardioprotection and nephroprotection) Antioxidant	[51,52,53,54,55,56,57]
*Tecoma stans* (L.) Juss. ex Kunth (Bignoniaceae)	Indigenous	Leaves	Aqueous	Antihyperlipidemic Antihyperglycemic antioxidant α-glucosidase inhibitor Antiglycation	[58,59,60,61,62]
*Momordica charantia* L. (Cucurbitaceae)	Exotic	Fruit	Aqueous Methanolic Ethanolic	Antihyperglycemic Antihyperlipidemic Antioxidant DM-associated complication protection (nephroprotection)	[63,64,65,66,67,68,69]
*Phyllanthus niruri* L. (Phyllanthaceae)	Exotic	Aerial parts Leaves	Ethanolic Aqueous	Antihyperlipidemic Antihyperglycemic Antiglycation DM-associated complication protection (nephroprotection) Antioxidant	[39,70,71]
